# Progress in Surgery

**Published:** 1898-04-09

**Authors:** 


### Progress in Surgery.

### GENITO-URINARY SURGERY.

**The Blood in Renal Disease.**—In the Goulstonian Lectures Dr. Rose Bradford summarises his conclusions as follows: (1) In suppression there is a great increase in the nitrogenous extractives in the blood and tissues; (2) in acute uræmia without dropsy there may be a still greater increase; (3) in chronic renal disease fatal from other causes, there is some increase, but less; (4) in renal disease, even in apparent good health, there is a small excess; and (5) in eclampsia the excess is small in amount.[1]

**Lavage in Genito-urinary Disease.**—Copious syringing of the bladder and urethra with permanganate of potash is now largely practised for evacuation of suspended substances in the urine, for local treatment of the vesical and urethral wall, and in other conditions. The solutions used are boiled water with or without sedatives, astringents, &c., in solution, salicylic and chromic acids, biniodide of mercury, formalin, and nitrate of silver. A saturated (4 per cent.) solution of boric acid is most commonly employed.

**Hydronephrosis.**—Two cases of this condition in children have been described by Mr. Owen; one was of congenital and the other of traumatic origin. The former was a girl of thirteen; the tumour was about the size of a fœtal head, and consisted of a dilated renal pelvis, with kidney substance stretched out in the wall of the cyst. The latter was in a boy of seven, who had been kicked by a horse, becoming immediately blanched and complaining of great pain. Three weeks afterwards a tumour appeared in the loin. This was aspirated, and bloody urine came away. Later, an exploratory incision was made, and the kidney was found torn right across. With drainage the boy recovered perfectly.[2]

**Sarcoma of Kidney.**—Three cases of sarcoma of the kidney in children are given by Dr. George Walker (Baltimore), with observations upon a collected series of nearly 150 cases. The three cases were pure sarcomata, occurring at the ages of 4, 6, and 13 respectively. Two died immediately after the operation, and one lived four months.

It was not until quite recently that the majority of malignant tumours of the kidney were found to be sarcomatous and not carcinomatous. As regards ætiology, traumatism appears to play the largest part. Clinically, the surrounding organs are displaced and pressed upon by the tumour, and the peritoneum usually contains a turbid brownish fluid. The colon is usually adherent to the front of the tumour. Adhesions to other surrounding structures are common. In no less than five cases, or one in thirty, there were adhesions to the inferior vena cava. The veins on the surface are commonly large. The weight of the tumour is sometimes great, weighing more than half that of the remaining child. The macroscopic section is variable, according to whether degeneration and hæmorrhage have occurred. The majority, of which there is adequate record, were of spindle and round-celled variety. Some were probably adenomatous, arising perhaps from remains of the Wolffian body. Metastases are most common in the liver, lungs, and opposite kidney. The four commonest symptoms are a tumour, pain, hæmaturia, and cachexia. The presence of a tumour is usually the first sign, and its growth is very rapid. The hæmaturia is intermittent, and in over 10 per cent. of cases is the initial symptom. Vomiting is the most constant pressure symptom, and death is usually due to exhaustion. Renal sarcomata are commonest between birth and the age of six; they are rare after eight. The left kidney is more often affected than the right.

Operation for this condition is very unfavourable, the operative mortality being at least 50 per cent., but statistics have apparently improved of late. Of the whole series of cases only four have exceeded the three years' limit for recurrence. If, therefore, operation is to be done it should be done early.[3]

**Floating Kidney.**—"Anchoring" the kidney is the method recommended by Dr. Harvey Reed (Columbus) for this condition. It is performed by making an abdominal section, and passing a suture through the upper part of the kidney and through the parietes, bringing the ends out at the back between the eleventh and twelfth ribs, where they are tied over a piece of gauze and left in for ten days.

An interesting condition of floating kidney, associated with a floating gall-bladder with torsion, is recorded by Dr. A. V. Wendle. The gall-bladder, which contained stinking pus and gall-stones, was removed, and the patient was soon convalescent.[4]

[1] Lancet, March 25, 1898. [2] Lancet, Dec. 4, 1897. [3] Annals of Surgery, Nov., 1897. [4] Annals of Surgery, Feb., 1898.

### HERNIA.

**Varieties of Hernia.**—Distinction is made between "intestinal" or inter-muscular hernia, and "properitoneal" hernia, the latter being that form of reduction *en masse* where the intestine lies between the abdominal wall and the peritoneum. An illustration of the parts of a properitoneal hernia into a diverticulum of the sac is given by Dr. Stone Scott.

Mr. C. B. Lockwood gives his experiences of 180 cases of supposed hernia, in some of which other conditions, *e.g.*, lipomata, varicocele, were found. A certain amount of subperitoneal fat normally descends with the testicle along the inguinal canal, and, together with varicose veins may be an inducing cause of hernia.[1]

**New Methods.**—Lannelongue and Demars have employed injections of zinc chloride with some success. Dr. Swift (U.S. Army), when doing Bassini's operation completes it by causing the upper and lower parts of the divided external oblique to overlap and sew the parts *in situ*.

Dr. S. R. Fowler has paid much attention to the subject, and after trial of several methods has devised a plan whereby the cord is transplanted within the peritoneum and the whole inguinal canal is totally closed.[2] With the patient in the Trendelenburg position an incision is made with its convexity downwards in the line of Poupart's ligament from the position of the internal ring to the spine of the pubis. The flap is reflected upwards. The external oblique is slit up and the cord and the sac are isolated, the latter being divided close. The transversalis fascia is then opened, and the deep epigastric vessels are ligatured and divided. The finger is now placed in the peritoneal cavity as a guide, and the whole posterior wall of the inguinal canal divided with scissors, and the cord placed in the peritoneal cavity, coming forwards only at the internal part. The tissues are then sewn up layer by layer. Six cases are recorded.

**Special Cases.**—Hernia was associated with hernia of the bladder in a case under Mr. T. Bates;[3] the bladder was opened, but sewn up again without untoward result.

Two cases are given by Mr. E. T. Milner in which Murphy's button was used. One was a femoral hernia, and recovered after resection; the other was inguinal, and died. As the superficial structures, including the skin, in this fatal case were gangrenous, it is doubtful whether resection was here expedient.[4]

Mr. Tubby describes some unusual cases. One was a congenital, left, inguinal hernia, and contained the cæcum appendix, &c. Another with similar contents occurred on the right side. Another case contained both tubes and ovaries in a hernia on the left side.[5]

**Umbilical Hernia.**—A specimen of strangulated umbilical hernia in a child five days old was shown by Mr. W. G. Spencer at the Pathological Society of London. It contained the cæcum and sloughing ileum, and the general opinion at the meeting was that the hernia was acquired and not congenital.[6]

[1] Lancet, Nov. 13, 1897. [2] Annals of Surgery, Nov. 1897. [3] Lancet, Oct. 9, 1897. [4] Lancet, Dec. 11, 1897. [5] B.M.J., Oct 9, 1897. [6] Lancet, Feb. 5, 1898.

